# Supplementary material for: Sub-diurnal asymmetric warming has amplified atmospheric dryness since the 1980s
Source: Nat Commun. 2025 Sep 9;16:8247. doi: 10.1038/s41467-025-63672-z (PMC12420828; doi:10.1038/s41467-025-63672-z)
Supplement: Supplementary file 1 — Supplementary Information [file 41467_2025_63672_MOESM1_ESM.pdf]

Supplementary Material for

Sub-diurnal asymmetric warming has amplified atmospheric dryness since the 1980s

Ziqian Zhong[1], Hans W. Chen[1,*], Aiguo Dai[2], Tianjun Zhou[3], Bin He[4], Bo Su[4, 5, 6]

[1]Department of Space, Earth and Environment, Division of Geoscience and Remote Sensing, Chalmers University of Technology, SE-412 96 Gothenburg, Sweden

[2]Department of Atmospheric and Environmental Sciences, University at Albany, State University of New York, Albany, NY, 12222, USA.

[3]State Key Laboratory of Numerical Modeling for Atmospheric Sciences and Geophysical Fluid Dynamics, Institute of Atmospheric Physics, Chinese Academy of Sciences, Beijing, 100029, China

[4]State Key Laboratory of Earth Surface Processes and Resource Ecology, Faculty of Geographical Science, Beijing Normal University, Beijing 100875, China.

[5]Regional Climate Group, Department of Earth Sciences, University of Gothenburg, S-40530 Gothenburg, Sweden.

[6]Stockholm Resilience Centre, Stockholm University, 10691 Stockholm, Sweden.

*e-mail: hans.chen@chalmers.se

This PDF file includes:

Supplementary Discussion 1

Supplementary Figures 1 to 28

Supplementary Discussion 1

ERA5-Land reanalysis data and HadISD observations.

Most of the analyses in our study are based on both HadISD[1] station data and the ERA5-Land reanalysis[2], aiming to provide complementary insights and enhance the robustness of our conclusions. This approach is motivated by the fact that both station observations and reanalysis products have their respective strengths and limitations. Since the spatial distribution of observation stations is highly uneven, averaging station data can be difficult to interpret across large regions. Recent studies have shown that the ERA5 reanalysis accurately represents diurnal variations in climatic variables, including air temperature and relative humidity[3,4], supporting the reliability of ERA5-Land, which uses ERA5 as meteorological forcing. Here, we compared ERA5-Land reanalysis data and co-located HadISD observations across 1398 stations/grid cells (Supplementary Fig. 1a). The monthly values of saturated vapor pressure (SVP), actual vapor pressure (AVP), vapor pressure deficit (VPD), daily maximum temperature (T_{max}), daily minimum temperature (T_{min}), and daily mean temperature (T_{mean}) from ERA5-Land all show strong correlations with HadISD observations, with median correlation coefficients exceeding 0.96 across all stations.

DTR from ERA5-Land showed slightly lower agreement with HadISD, with a median correlation of 0.85. Spatially, stations in Europe and North America exhibited higher correlations between ERA5-Land and HadISD DTR (Supplementary Fig. 1b), consistent with regional studies reporting good performance of ERA5(-Land) air temperature[5,6]. However, stations in East Asia showed lower correlations. Previous studies in China suggest that ERA5-Land temperature biases tend to increase with elevation[7], especially in mountainous areas of western China[8,9]. This bias is primarily attributed to elevation differences between ground-based observation stations and corresponding ERA5-Land grid points[8,9]. This indicates that local conditions—such as complex terrain—may exert a significant influence at these stations, which cannot be fully captured by the relatively coarse spatial resolution of ERA5-based reanalysis datasets[10]. Additionally, the lower correlation observed in East Asia may also be attributed to sparser station coverage. As noted in

previous studies, while ERA5-based reanalysis generally show good consistency across most global regions, uneven station density can lead to reduced accuracy in data-sparse areas[11].

For the period prior to 1980, previous studies have suggested that both Australia[12] and China[13] exhibit pronounced discrepancies between ERA5 surface temperature and observational climate records during the 1960s. These discrepancies are due to the limited availability of input observations and reanalysis capabilities in the early years. Consequently, ERA5-Land—which uses ERA5 as its meteorological forcing—may inherit similar regional temperature biases before 1980s.

Supplementary Figures

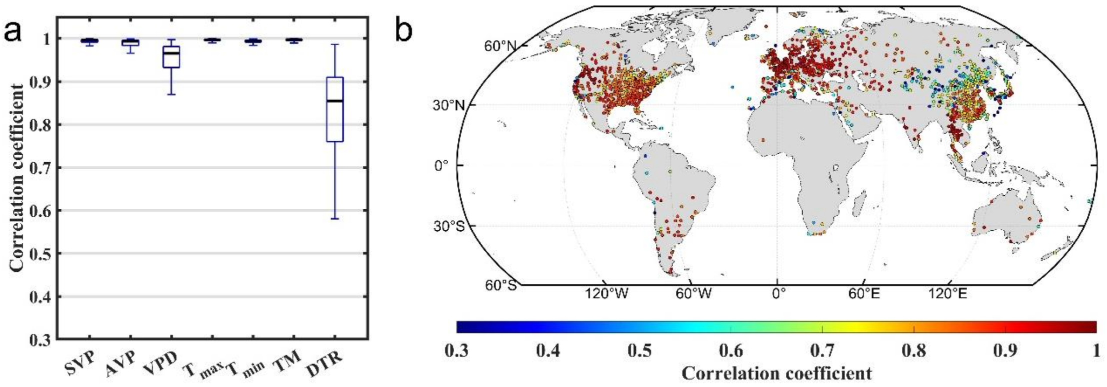

Supplementary Figure 1. Correlation between temperature and humidity variables derived from station observations and ERA5-Land data during 1980–2023. a, Boxplots of the Pearson correlation coefficients between monthly averages of saturated vapor pressure (SVP), actual vapor pressure (AVP), vapor pressure deficit (VPD), daily maximum temperature (T_{max}), daily minimum temperature (T_{min}), daily mean temperature (T_{mean}), and diurnal temperature range (DTR), calculated from HadISD station observations and their nearest ERA5-Land grid cells across 1398 stations. The height of each box represents the interquartile range, with the thick black line indicating the median and the box edges denoting the first and third quartiles. Whiskers extend to the 2.5th and 97.5th percentiles. **b,** Spatial distribution of the Pearson correlation between monthly station-based and ERA5-Land-derived DTR across stations.

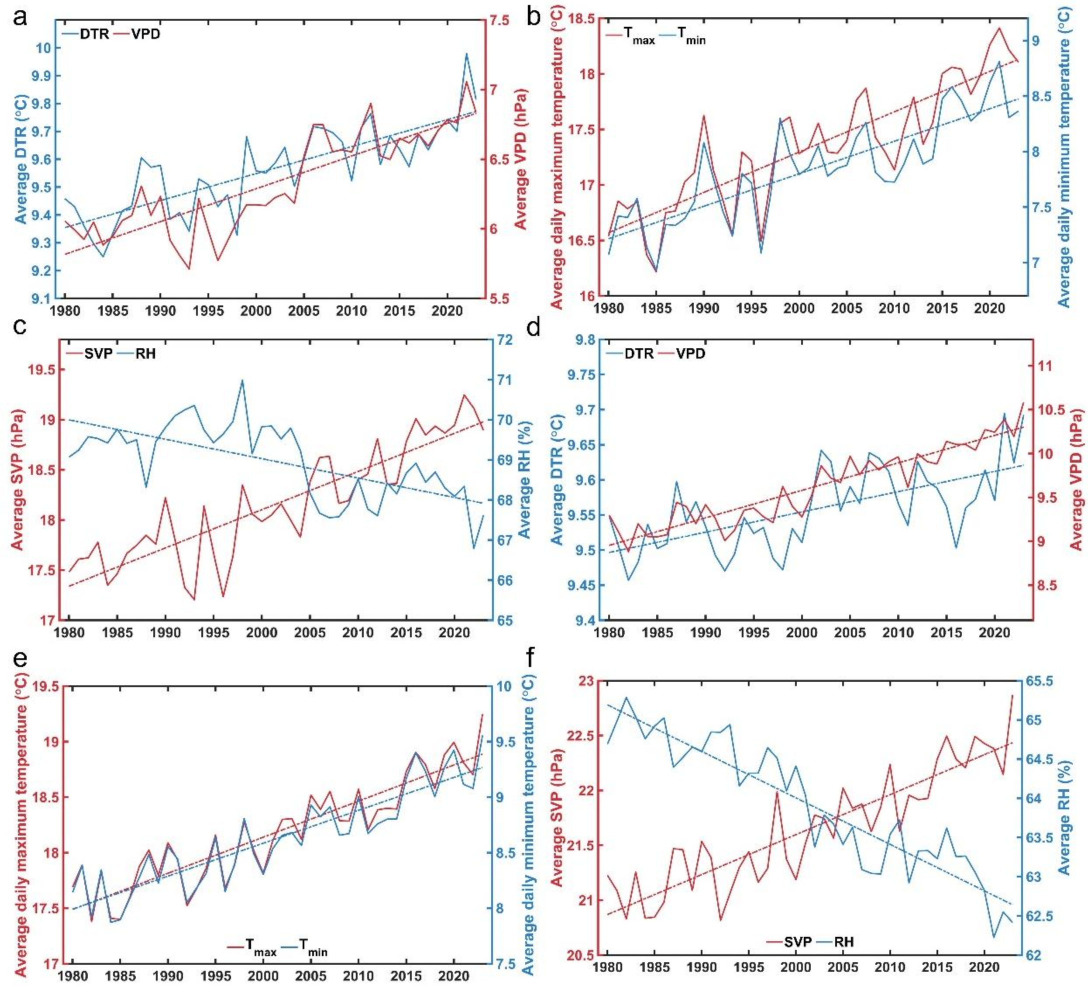

Supplementary Figure 2. Variations and changes in annual average saturated vapor pressure (SVP), relative humidity (RH), daily maximum temperature (T_{max}), daily minimum temperature (T_{min}), vapor pressure deficit (VPD) and diurnal temperature range (DTR) across all stations or over land during 1980–2023. a-c, Variations and changes in annual average DTR and VPD (**a**), SVP and RH (**b**) and T_{max} and T_{min} (**c**) across all stations. **d–f**, Variations and changes in annual area-weighted average DTR and VPD (**d**), T_{max} and T_{min} (**e**), and SVP and RH (**f**) derived from the ERA5-Land dataset. The dashed lines show the linear trends obtained from linear regressions.

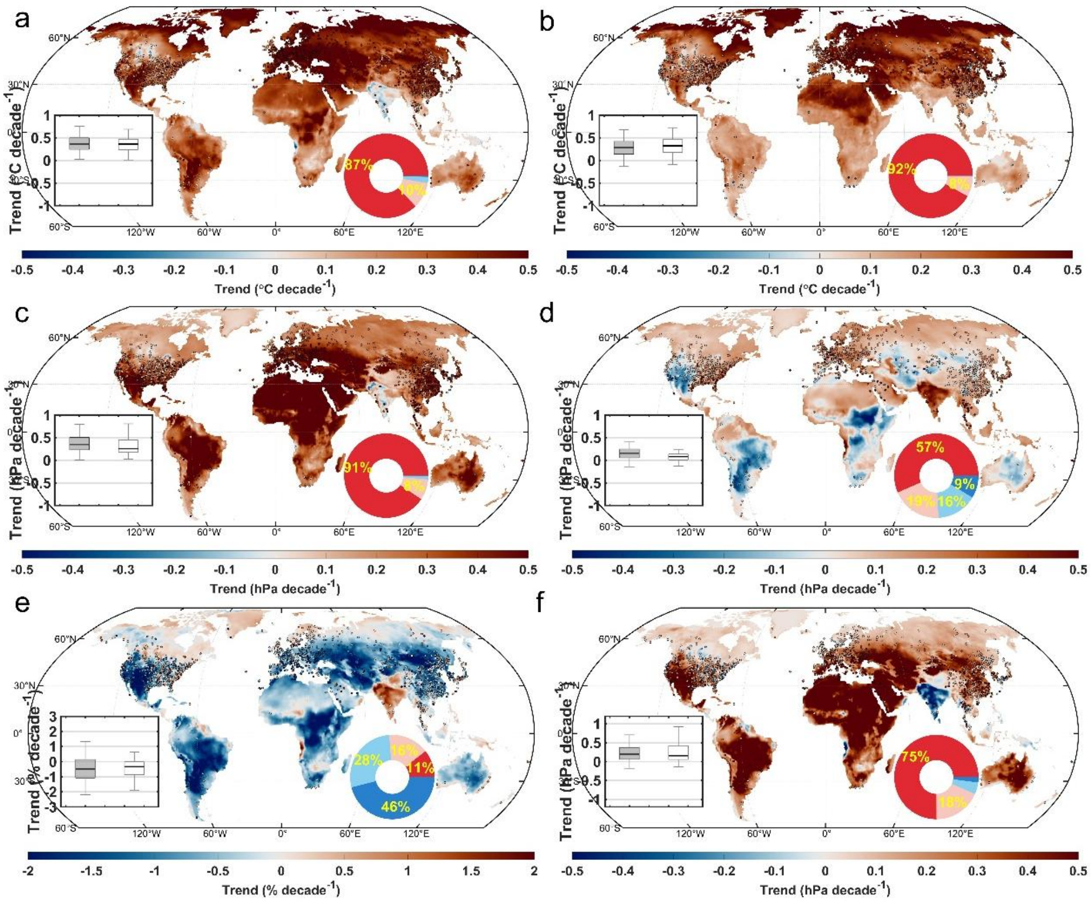

Supplementary Figure 3. Spatial distribution of the trend in temperature and humidity variables during 1980–2023. Trend in annual average daily maximum temperature (**a**), daily minimum temperature (**b**), saturated vapor pressure (**c**), actual vapor pressure (**d**), relative humidity (**e**), and vapor pressure deficit (**f**) over land areas during 1980–2023**.** Insets show boxplots of trends across observation stations (solid boxes) and ERA5-Land grid points (hollow boxes). Pie charts show the percentage of land area with significantly positive (red), weak positive (light red), weak negative (light blue), and significantly negative (blue) trends ($p < 0.05$), based on ERA5-Land data. In all boxplots, the height of each box represents the interquartile range of trends across different stations or grid points, with the thick black line indicating the median, and the edges denoting the first and third quartiles. Whiskers extend to the 2.5th and 97.5th percentiles.

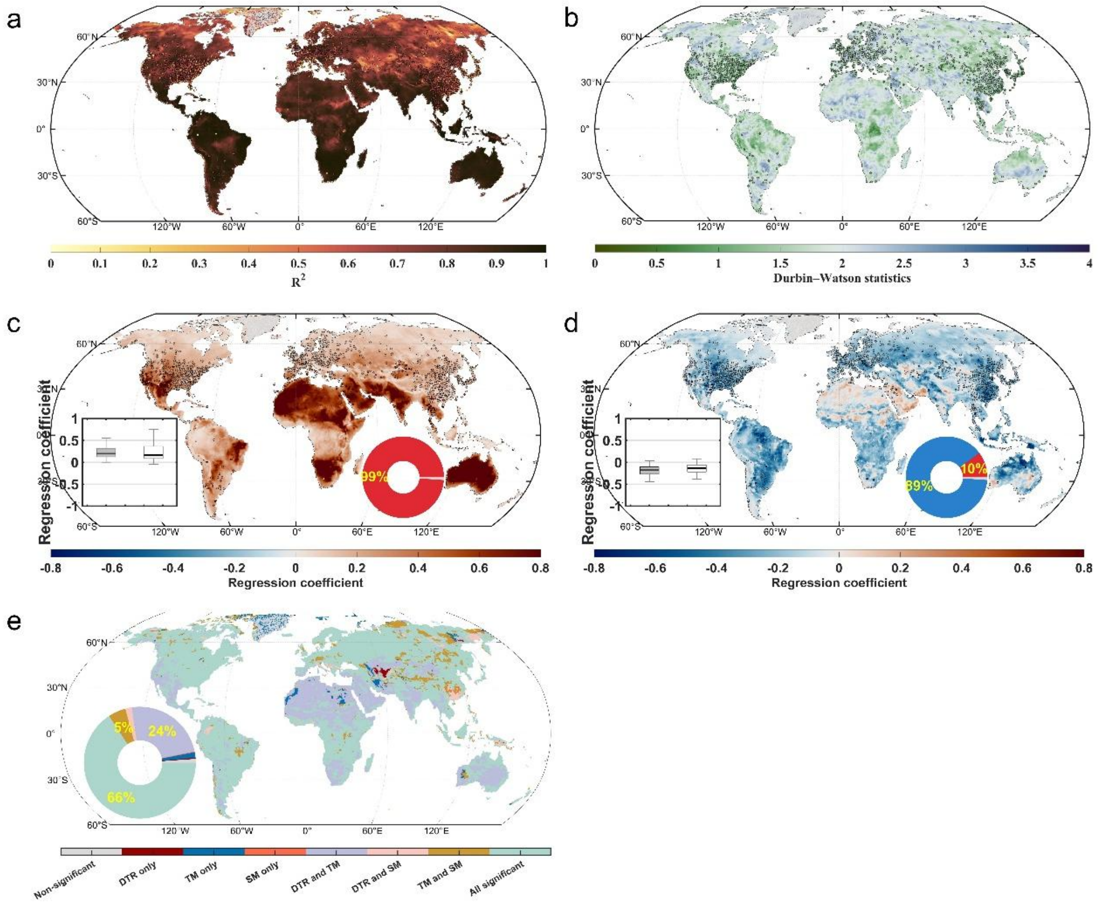

Supplementary Figure 4. Impact of mean air temperature (T_{mean}) and soil moisture (SM) on vapor pressure deficit (VPD) on the interannual scale during 1980–2023. a-b, Spatial distribution of determination coefficients (R^2, **a**) and Durbin–Watson statistic from the ridge regression (RR) model defined in Equation (2). **c-d**, Spatial distribution of RR coefficients of detrended annual VPD with respect to T_{mean} (**c**) and SM (**d**). Insets show boxplots of RR coefficients across observation stations (solid boxes) and grid points (hollow boxes). Pie charts show the percentage of land area with positive (red), negative (blue), and non-significant (light grey) RR coefficients based on ERA5-Land data. "Non-significant" refers to cases where none of the coefficients in the RR model are statistically significant. **e**, Spatial distribution of significant driver combinations. Each grid cell is classified according to the combination of independent variables—diurnal temperature range (DTR), T_{mean}, and SM—that significantly contribute to interannual variability in the dependent variable. Pie chart shows the percentage of land area associated with each significant driver combination based on ERA5-Land data. All variables in the regressions are detrended and standardized annual averages. Stations (1.07%) and areas (1.1%) with non-significant RR coefficients are masked in light grey and excluded from the analysis. In all boxplots, the height of each box represents the interquartile, with the thick black line indicating the median, and the edges denoting the first and third quartiles. Whiskers extend to the 2.5th and 97.5th percentiles.

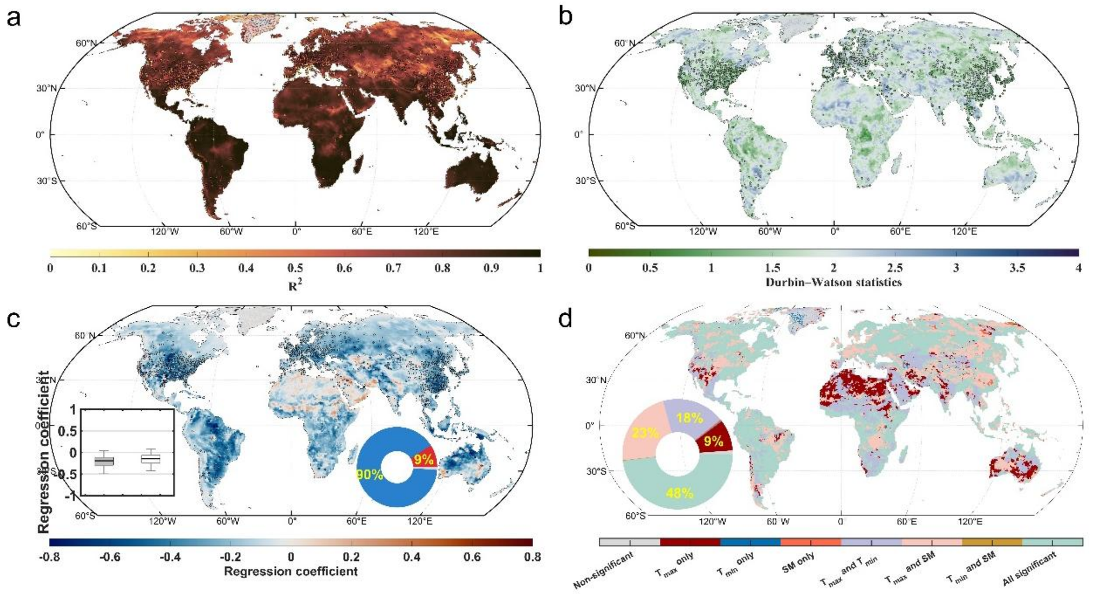

Supplementary Figure 5. Impact of soil moisture (SM) on vapor pressure deficit (VPD) on the interannual scale during 1980–2023. a-b, Spatial distribution of determination coefficients (R^2, **a**) and Durbin–Watson statistic from the ridge regression (RR) model defined in Equation (3). **c**, Spatial distribution of RR coefficients of detrended annual VPD with respect to SM. Inset shows boxplot of RR coefficients across observation stations (solid boxes) and grid points (hollow boxes). Pie chart shows the percentage of land area with positive (red), negative (blue), and non-significant (light grey) RR coefficients based on ERA5-Land data. "Non-significant" refers to cases where none of the coefficients in the RR model are statistically significant. **d**, Spatial distribution of significant driver combinations. Each grid cell is classified according to the combination of independent variables—daily maximum (T_{max}) and minimum temperatures (T_{min}), and SM—that significantly contribute to interannual variability in the dependent variable. Pie chart shows the percentage of land area associated with each significant driver combination based on ERA5-Land data. All variables in the regressions are detrended and standardized annual averages. Stations (1.29%) and areas (1.11%) with non-significant RR coefficients are masked in light grey and excluded from the analysis. In all boxplots, the height of each box represents the interquartile, with the thick black line indicating the median, and the edges denoting the first and third quartiles. Whiskers extend to the 2.5th and 97.5th percentiles.

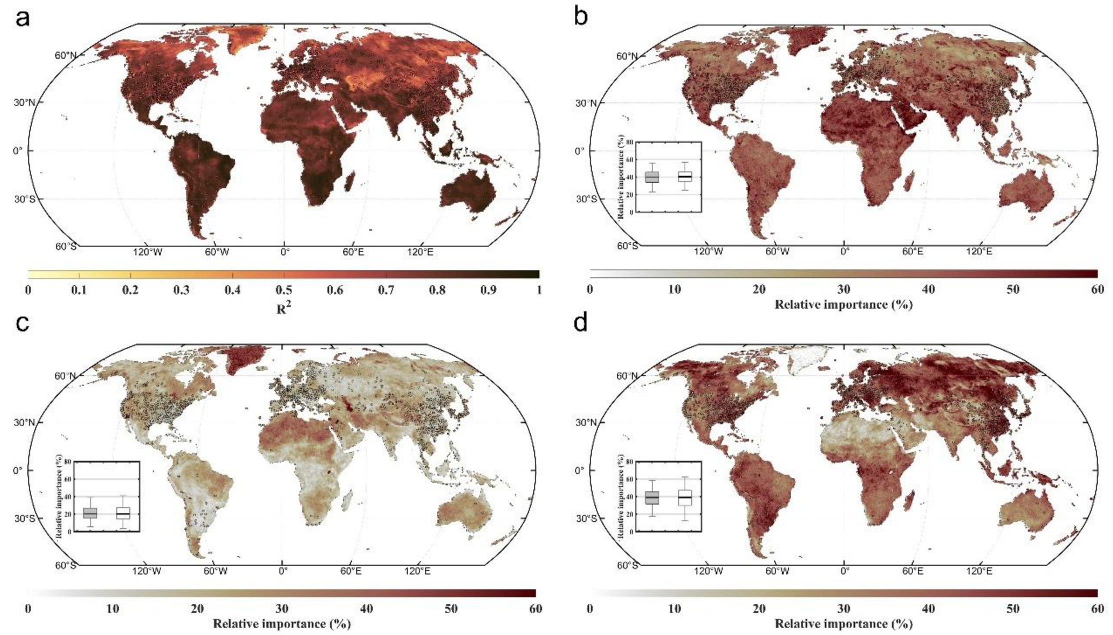

Supplementary Figure 6. Relative importance of daily maximum (T_{max}) and minimum temperatures (T_{min}) and soil moisture (SM) in vapor pressure deficit (VPD) on the interannual scale during 1980–2023. a, Spatial distribution of determination coefficients (R^2) from the random forest (RF) regression model defined in Equation (3). **b-d**, Spatial distribution of relative importance of T_{max} (b), T_{min} (c) and SM (d) in driving interannual VPD variability, identified using the RF regression model with the Shapley Additive Explanations framework (SHAP) framework. Insets show boxplot of relative importance (%) across observation stations (solid boxes) and ERA5-Land grid points (hollow boxes). All variables in the regressions are detrended and standardized annual averages. In all boxplots, the height of each box represents the interquartile, with the thick black line indicating the median, and the edges denoting the first and third quartiles. Whiskers extend to the 2.5th and 97.5th percentiles.

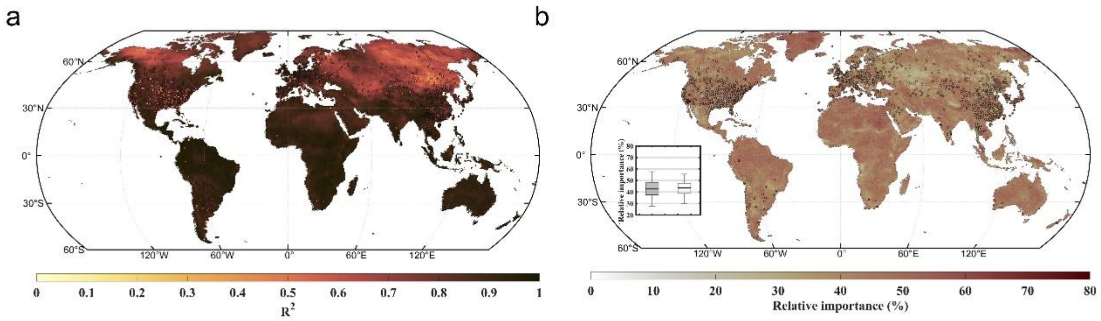

Supplementary Figure 7. Relative importance of daily maximum (T_{max}) and minimum temperatures (T_{min}) in driving saturation vapor pressure (SVP) on the interannual scale during 1980–2023. a, Spatial distribution of determination coefficients (R^2) from the random forest regression (RF) regression model defined in Equation (4). **b**, Spatial distribution of relative importance of T_{min} in driving interannual SVP variability, identified using the RF regression model with the Shapley Additive Explanations framework (SHAP) framework. Inset shows boxplot of relative importance (%) across observation stations (solid boxes) and ERA5-Land grid points (hollow boxes). All variables in the regressions are detrended and standardized annual averages. In all boxplots, the height of each box represents the interquartile, with the thick black line indicating the median, and the edges denoting the first and third quartiles. Whiskers extend to the 2.5th and 97.5th percentiles.

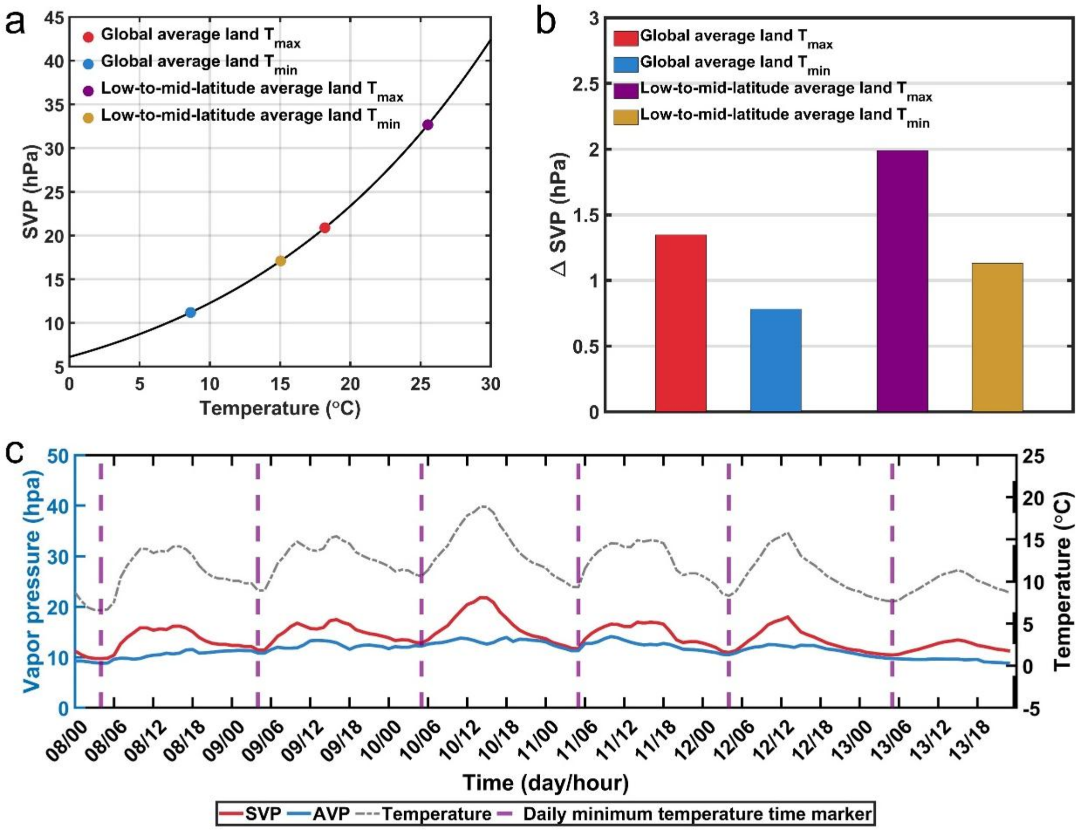

Supplementary Figure 8. The impact of daily maximum temperature (T_{max}) and daily minimum temperature (T_{min}) on saturated vapor pressure (SVP) and actual vapor pressure (AVP). a, The relationship between SVP and temperature. The points on the curve indicate the global area-weighted average land T_{max} and T_{min}, as well as the low-to-mid-latitude (between 45°S and 45°N) area-weighted average land T_{max} and T_{min} during 1980–2023. **b**, Changes in SVP (ΔSVP) due to a 1°C increase at different temperature conditions indicated in (a). **c**, Demonstration of how T_{min} limits the changes in AVP. The graph shows variations in vapor pressure (SVP and AVP; left y-axis) and temperature (right y-axis) at 45°N, 5°E from May 8 to May 13, 2010 (UTC time). Despite fluctuations in temperature and SVP, AVP is constrained by the T_{min} values during this period. All temperature and humidity variables were derived from the ERA5-Land dataset.

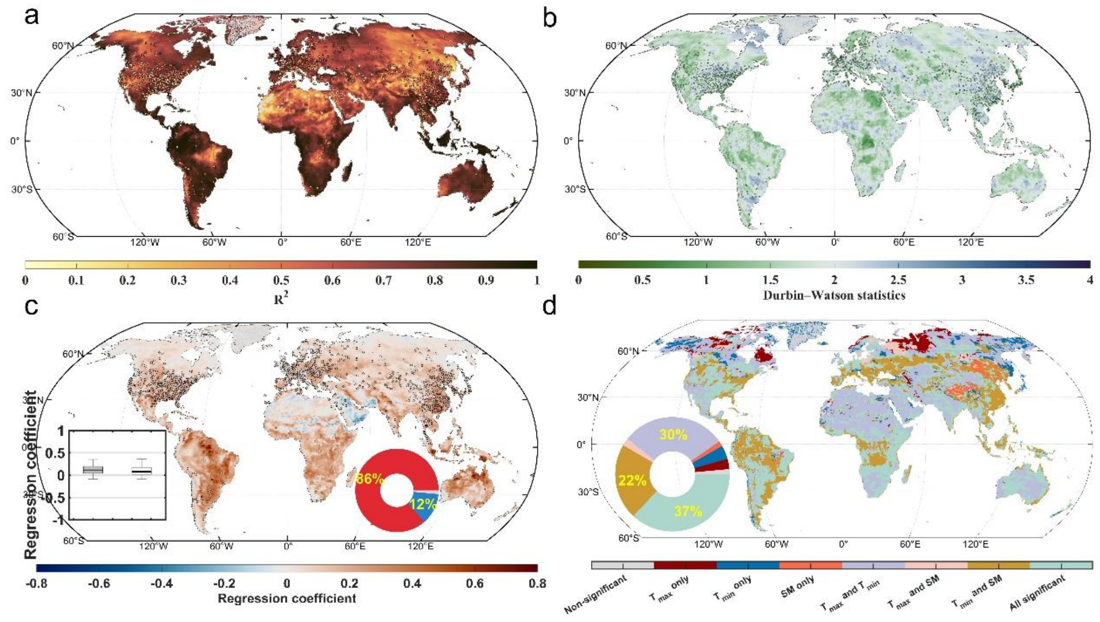

Supplementary Figure 9. Impact of soil moisture (SM) on actual vapor pressure (AVP) on the interannual scale during 1980–2023. a-b, Spatial distribution of determination coefficients (R^2, **a**) and Durbin–Watson statistic from the ridge regression (RR) model defined in Equation (5). **c**, Spatial distribution of RR coefficients of detrended annual AVP with respect to SM. Inset shows boxplot of RR coefficients across observation stations (solid boxes) and grid points (hollow boxes). Pie chart shows the percentage of land area with positive (red), negative (blue), and non-significant (light grey) RR coefficients based on ERA5-Land data. "Non-significant" refers to cases where none of the coefficients in the RR model are statistically significant. **d**, Spatial distribution of significant driver combinations. Each grid cell is classified according to the combination of independent variables—daily maximum (T_{max}) and minimum temperatures (T_{min}) and SM—that significantly contribute to interannual variability in the dependent variable. Pie chart shows the percentage of land area associated with each significant driver combination based on ERA5-Land data. All variables in the regressions are detrended and standardized annual averages. Stations (4.36%) and areas (1.45%) with non-significant RR coefficients are masked in light grey and excluded from the analysis. In all boxplots, the height of each box represents the interquartile, with the thick black line indicating the median, and the edges denoting the first and third quartiles. Whiskers extend to the 2.5th and 97.5th percentiles.

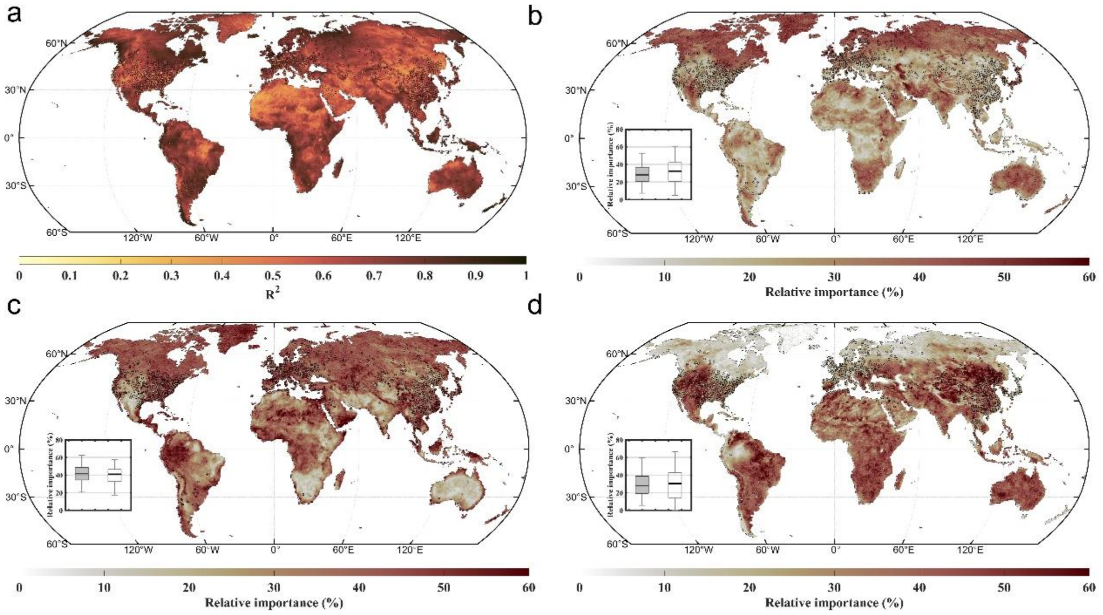

Supplementary Figure 10. Relative importance of daily maximum (T_{max}) and minimum temperatures (T_{min}) and soil moisture (SM) in actual vapor pressure (AVP) on the interannual scale during 1980–2023. a, Spatial distribution of determination coefficients (R^2) from the random forest regression (RF) regression model defined in Equation (5). **b-d**, Spatial distribution of relative importance of T_{max} (b), T_{min} (c) and SM (d) in driving interannual AVP variability, identified using the RF regression model with the Shapley Additive Explanations framework (SHAP) framework. Insets show boxplots of relative importance (%) across observation stations (solid boxes) and ERA5-Land grid points (hollow boxes). All variables in the regressions are detrended and standardized annual averages. In all boxplots, the height of each box represents the interquartile, with the thick black line indicating the median, and the edges denoting the first and third quartiles. Whiskers extend to the 2.5th and 97.5th percentiles.

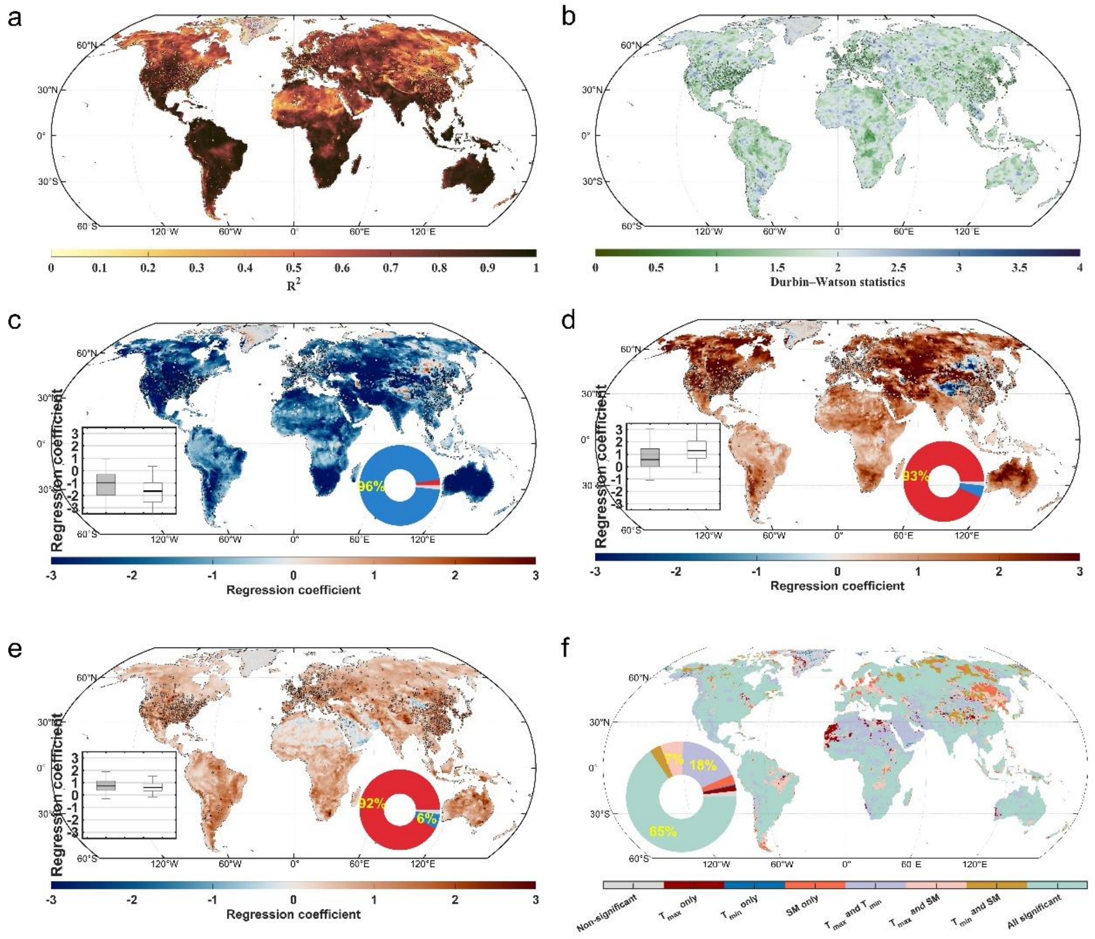

Supplementary Figure 11. Impact of daily maximum (T_{max}) and minimum temperatures (T_{min}) and soil moisture (SM) on relative humidity (RH) on the interannual scale during 1980–2023. **a-b**, Spatial distribution of determination coefficients (R^2, **a**) and Durbin–Watson statistic from the ridge regression (RR) model defined in Equation (6). **c-e**, Spatial distribution of RR coefficients of detrended annual VPD with respect to T_{max} (**c**), T_{min} (**d**) and SM (**e**). Insets show boxplots of RR coefficients across observation stations (solid boxes) and grid points (hollow boxes). Pie charts show the percentage of land area with positive (red), negative (blue), and non-significant (light grey) RR coefficients based on ERA5-Land data. "Non-significant" refers to cases where none of the coefficients in the RR model are statistically significant. **f**, Spatial distribution of significant driver combinations. Each grid cell is classified according to the combination of independent variables— that significantly contribute to interannual variability in the dependent variable. Pie chart shows the percentage of land area associated with each significant driver combination based on ERA5-Land data. All variables in the regressions are detrended and standardized annual averages. Stations (6.08%) and areas (0.77%) with non-significant RR coefficients are masked in light grey and excluded from the analysis. In all boxplots, the height of each box represents the interquartile, with the thick black line indicating the median, and the edges denoting the first and third quartiles. Whiskers extend to the 2.5th and 97.5th percentiles.

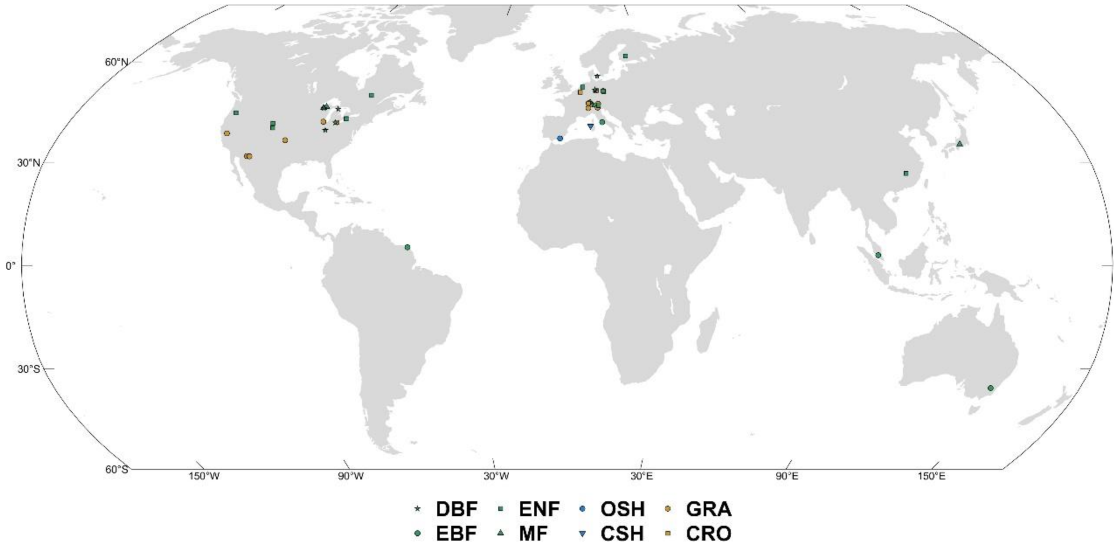

Supplementary Figure 12. Spatial distribution of FLUXNET tower sites. The biomes include evergreen needleleaf forest (ENF), evergreen broadleaf forest (EBF), deciduous broadleaf forest (DBF), mixed forest (MF), closed shrub (CSH), open shrub (OSH), grassland (GRA) and cropland (CRO).

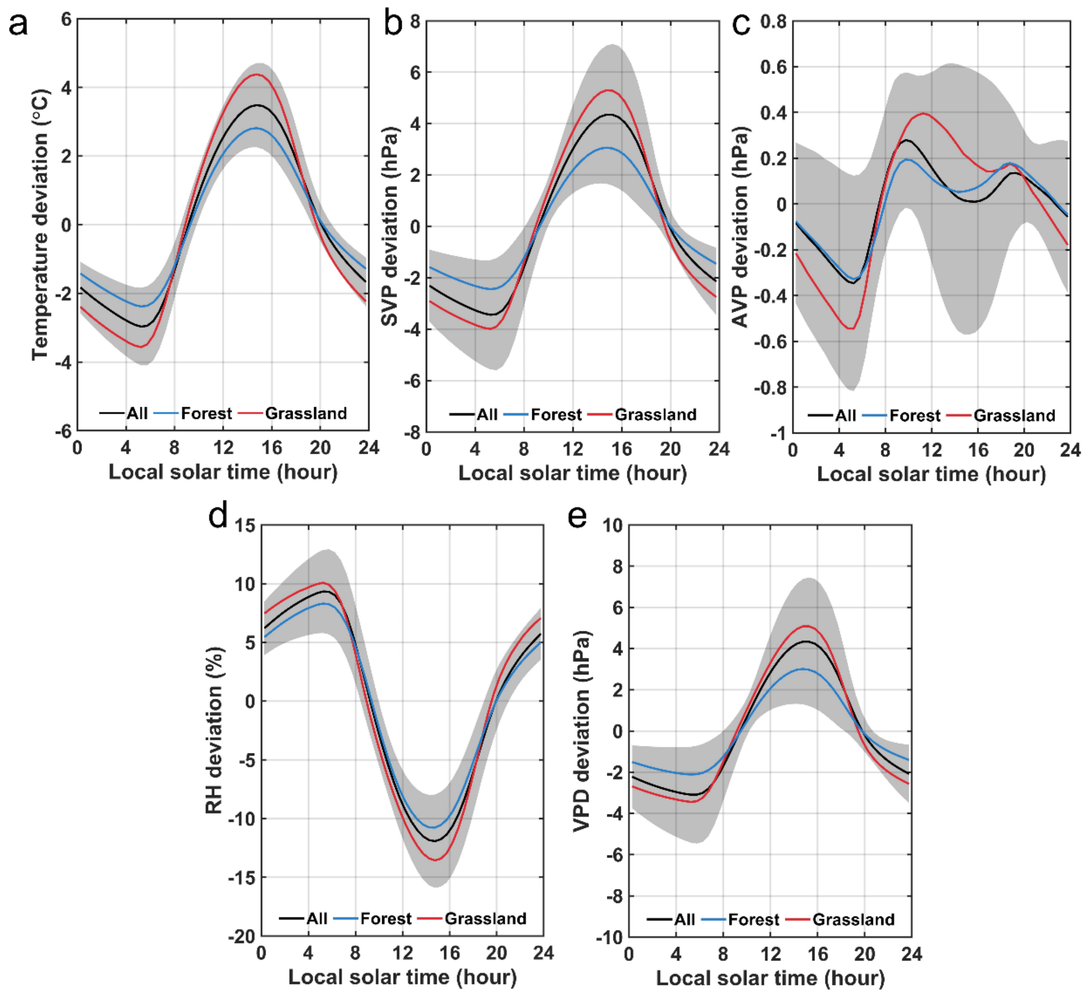

Supplementary Figure 13. Diurnal variations in temperature and humidity variables across FLUXNET tower sites. Averaged annual diurnal variations in temperature (**a**), saturated vapor pressure (SVP, **b**), actual vapor pressure (AVP, **c**), relative humidity (RH, **d**) and vapor pressure deficit (VPD, **e**) across all 56 sites. The y-axis represents the annual-average half-hourly variable deviations from the daily mean. The black thick lines represent the mean value and the shading the standard deviation around the mean for all sites. The blue and red thick lines represent the mean value for 32 forest sites and 12 grassland sites, respectively.

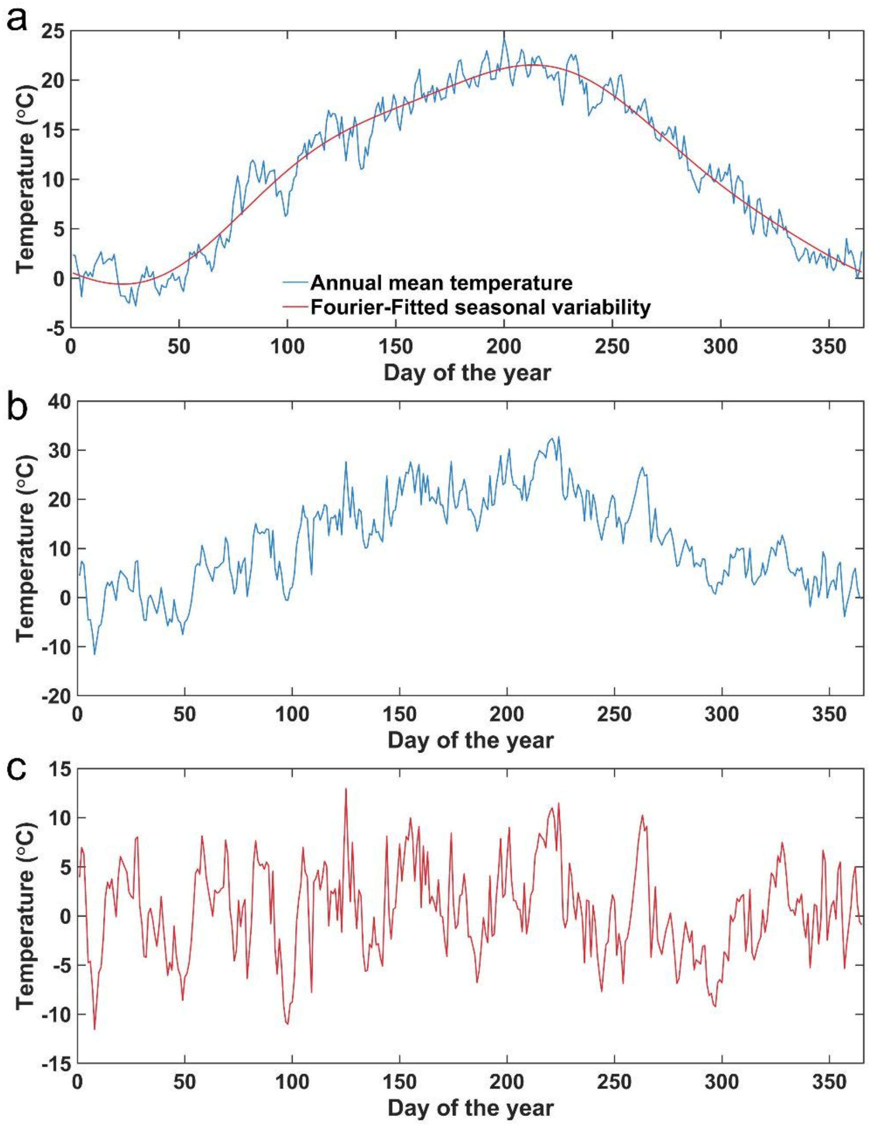

Supplementary Figure 14. Deseasonalization process for the seasonality of daily observations at FLUXNET sites. a, Mean daily maximum temperature across all years and its seasonal variation derived using Fast Fourier Transform. **b**, Daily maximum temperature observation series for the year 2003. **c**, Deseasonalized daily maximum temperature series for the year 2003 obtained after removing the seasonal cycle. The example station is Leinefelde (DE-Lnf), located at 51.33°N, 10.37°E.

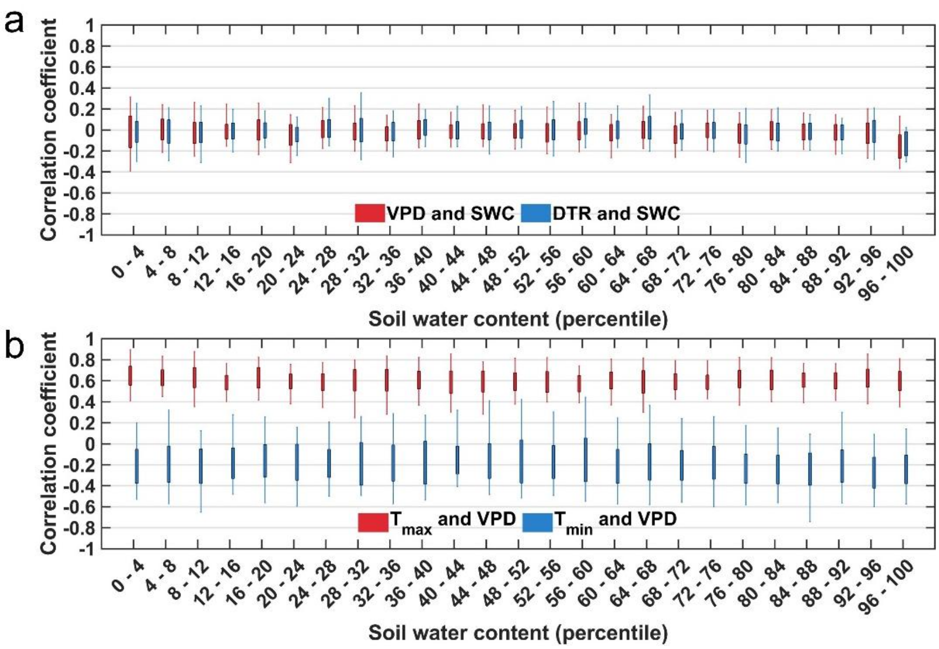

Supplementary Figure 15. Correlation between temperature and humidity variables across different soil water content (SWC) percentiles across FLUXNET sites. a, Assessment of the correlation between saturated SWC and vapor pressure deficit (VPD) or diurnal temperature range (DTR) across different soil water content percentiles at all stations. **b**, Assessment of the partial correlation between daily maximum temperature (T_{max}) or daily minimum temperature (T_{min}) and VPD while controlling for the other variable across different soil water content percentiles at all stations. The height of each box represents the interquartile range of correlation coefficients across different stations, with the edges denoting the first and third quartiles. Whiskers extend to the 2.5th and 97.5th percentiles of the correlation coefficient.

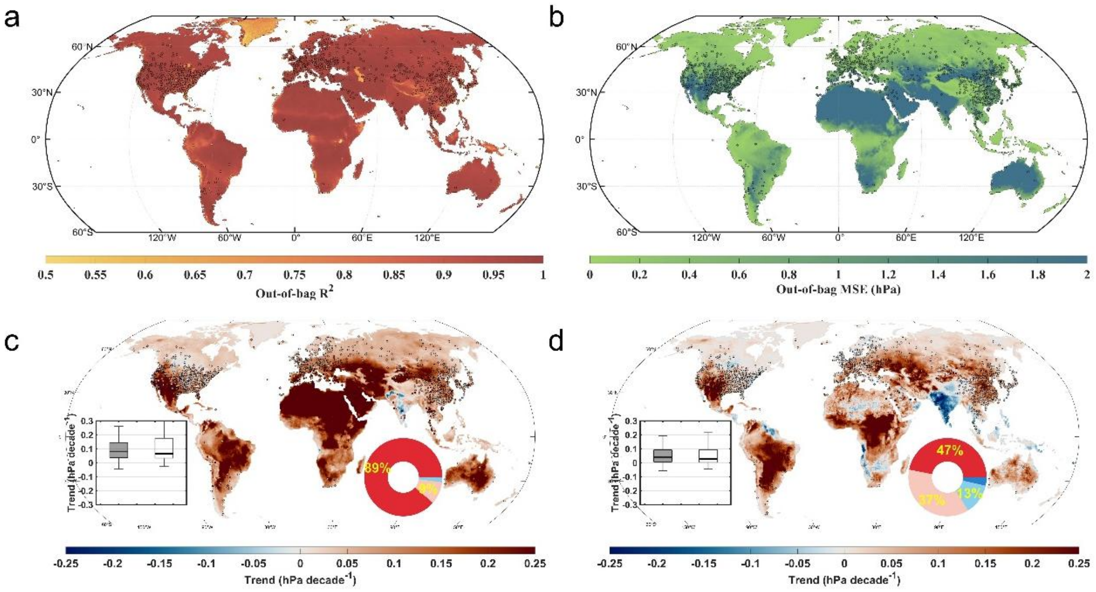

Supplementary Figure 16. Contribution of change in monthly air mean temperature (T_{mean}) and soil moisture (SM) to change in vapor pressure deficit (VPD) during 1980–2023. a-b, Spatial distribution of out-of-bag R-squared (OOB R^2, **a**) and out-of-bag mean squared error (OOB MSE, **b**) of the random forest regression model defined in Equation (2). **c-d**, Spatial distribution of trends in contribution of T_{mean} (**c**) and SM (**d**) to VPD change. Insets show boxplots of trends across observation stations (solid boxes) and ERA5-Land grid points (hollow boxes). Pie charts show the percentage of land area with significantly ($p < 0.05$) positive (red), weak positive (light red), weak negative (light blue), and significantly negative (blue) trends, based on ERA5-Land data. In all boxplots, the height of each box represents the interquartile range of trends or differences across different stations or grid points, with the thick black line indicating the median, and the edges denoting the first and third quartiles. Whiskers extend to the 2.5th and 97.5th percentiles.

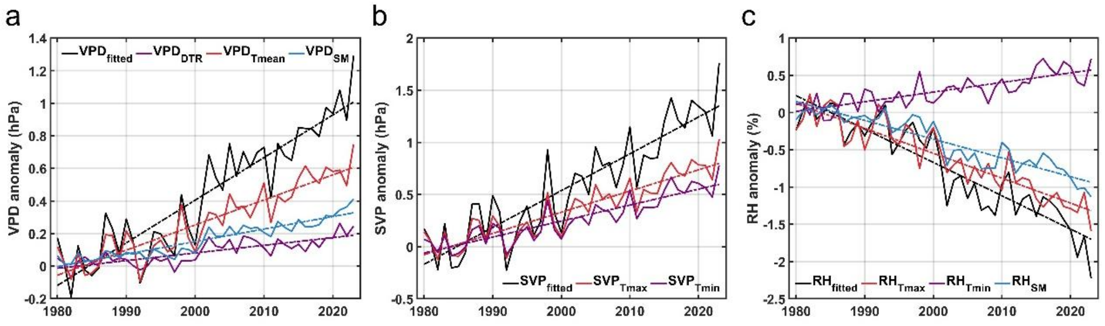

Supplementary Figure 17. Contribution of diurnal temperature range (DTR), daily mean temperature (T_{mean}), soil moisture (SM), daily maximum temperature (T_{max}) and daily minimum temperature (T_{min}) to trends in vapor pressure deficit (VPD), saturated vapor pressure (SVP) and relative humidity (RH) from 1980 to 2023 derived from the ERA5-Land dataset. Variations and changes in the annual average model-fitted (subscript fitted) VPD (**a**), SVP (**b**), and RH (**c**), and the contributions of T_{max} (subscript T_{rax}), T_{min} (subscript T_{min}), SM (subscript SM), DTR (subscript DTR) and T_{mean} (subscript T_{mean}) to the variations and changes over land. The model-fitted values are anomalies calculated by subtracting the mean values for the control period (1980–1982). The dashed lines show the linear trends obtained from linear regressions.

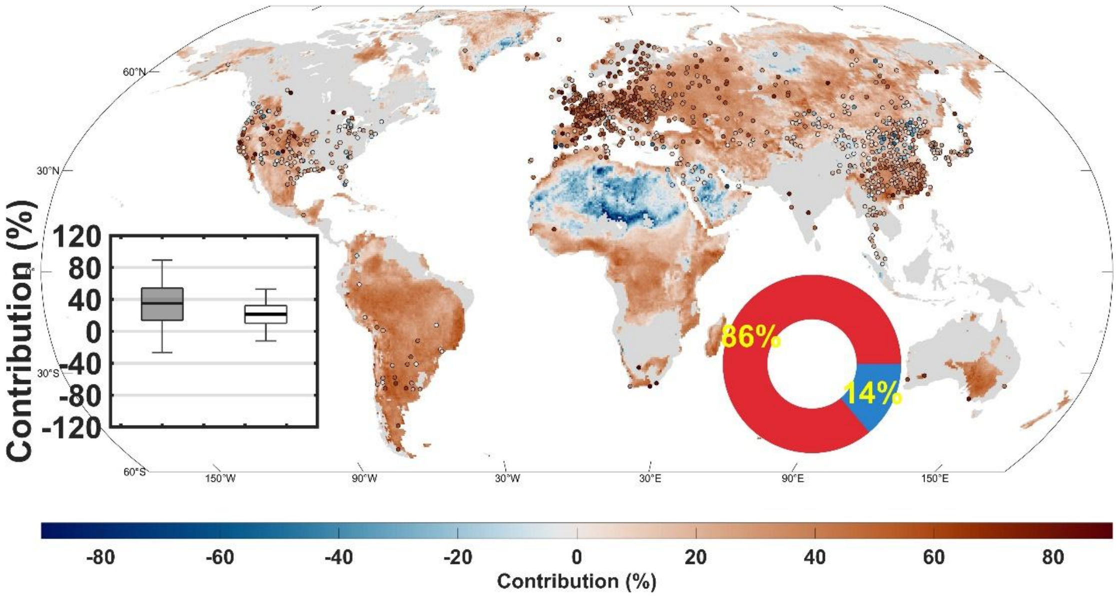

Supplementary Figure 18. Contribution rate of diurnal temperature range (DTR) change to vapor pressure deficit (VPD) increase during 1980–2023. The contribution rate is defined as the ratio of the trend in the contribution of DTR to VPD change to the trend in VPD. Inset show boxplot of contribution rate across stations (solid boxes) and ERA5-Land grid points (hollow boxes). The height of each box represents the interquartile range of contribution across different stations or grid points, with the thick black line indicating the median, and the edges denoting the first and third quartiles. Whiskers extend to the 2.5th and 97.5th percentiles. Pie chart shows the proportion of land area showing positive (red) or negative (blue) contributions, based on ERA5-Land data. Only sites (66.31%) or grid cells (68.46%) where model-simulated VPD exhibited a significant (p < 0.05) increase are included in the analysis and shown. Areas (31.54%) with either insignificant or negative model-simulated VPD trend are masked in light grey.

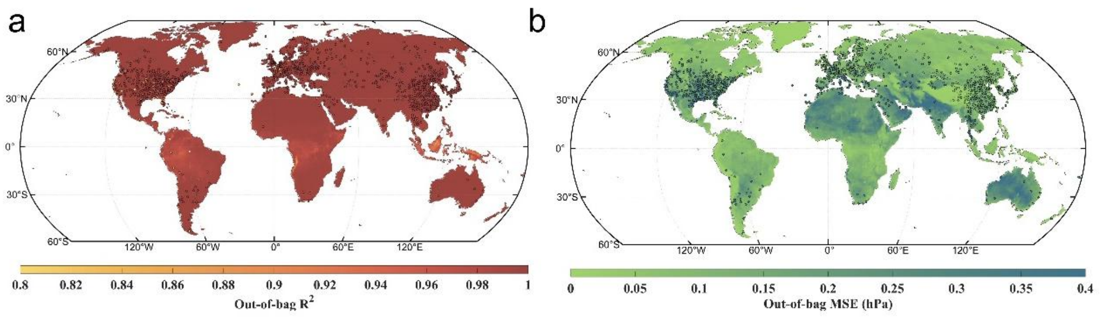

Supplementary Figure 19. Contribution of change in monthly sub-daily temperature to change in saturation vapor pressure (SVP) during 1980–2023. Spatial distribution of out-of-bag R-squared (OOB R^2, **a**) and out-of-bag mean squared error (OOB MSE, **b**) of the random forest regression model defined in Equation (4).

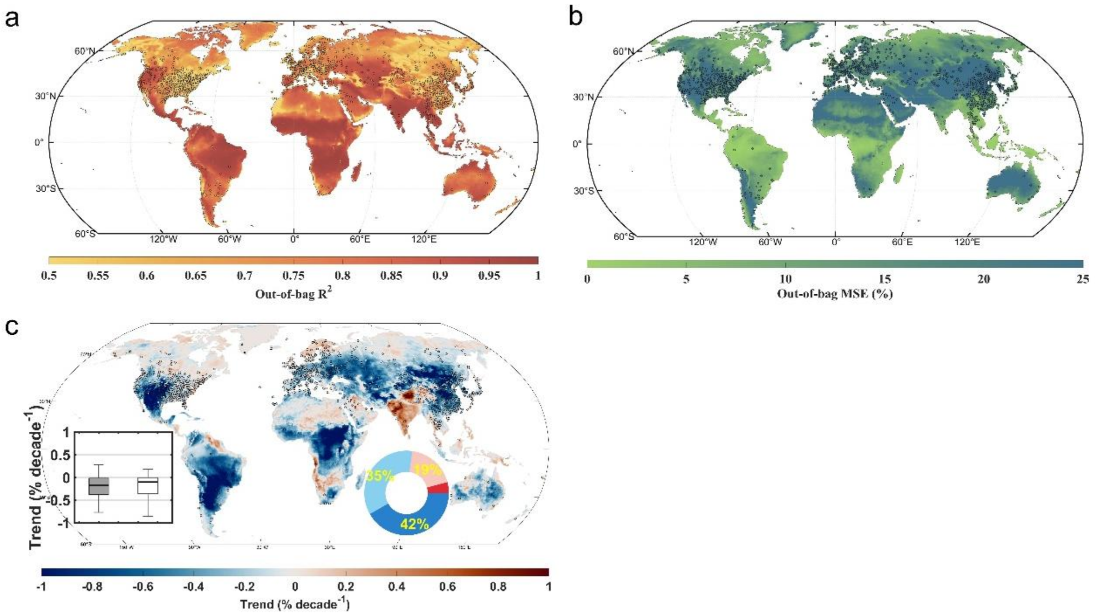

Supplementary Figure 20. Contribution of change in monthly soil moisture (SM) to change in relative humidity (RH) during 1980–2023. a-b, Spatial distribution of out-of-bag R-squared (OOB R^2, **a**) and out-of-bag mean squared error (OOB MSE, **b**) of the random forest regression model defined in Equation (6). **c**, Spatial distribution of trends in contribution of SM to RH change. Inset shows boxplot of trends across observation stations (solid boxes) and ERA5-Land grid points (hollow boxes). The height of each box represents the interquartile range of trends or differences across different stations or grid points, with the thick black line indicating the median, and the edges denoting the first and third quartiles. Whiskers extend to the 2.5th and 97.5th percentiles. Pie chart shows the percentage of land area with significantly ($p < 0.05$) positive (red), weak positive (light red), weak negative (light blue), and significantly negative (blue) trends, based on ERA5-Land data.

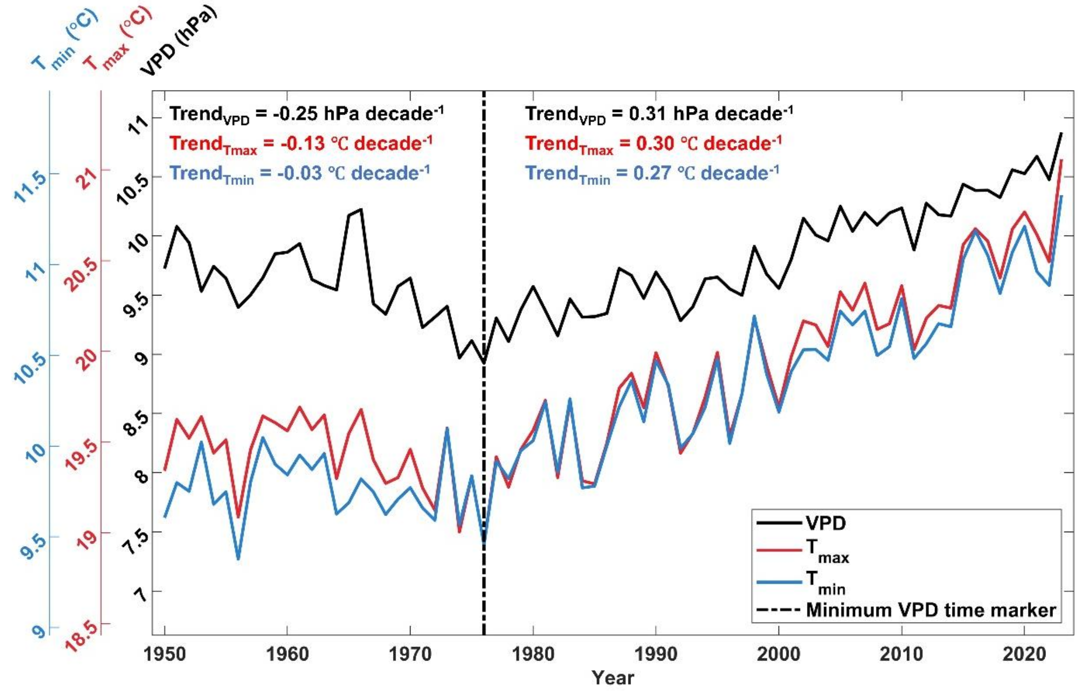

Supplementary Figure 21. Variations and trends in annual average vapor pressure deficit (VPD), daily maximum temperature (T_{max}) and daily minimum temperature (T_{min}) over land areas from 1950 to 2023, based on the ERA5-Land dataset. All trends in annual T_{max}, T_{min}, and VPD during 1950–1976 and 1977–2023 are statistically significant ($p < 0.05$), except for the trend in T_{min} during 1950–1976, which is not significant.

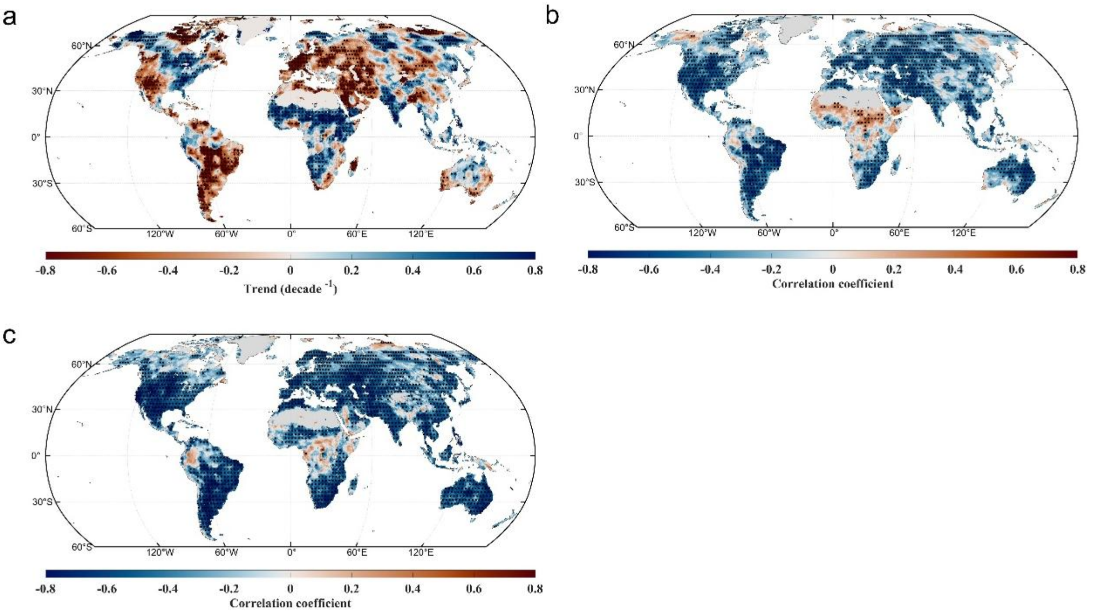

Supplementary Figure 22. Trend in the self-calibrated Palmer Drought Severity Index (scPDSI) and its correlation with VPD and DTR during 1980–2023. a, Spatial distribution of the trend in scPDSI over global land areas. **b–c,** Spatial distribution of the Pearson correlation between scPDSI and VPD (b) and between scPDSI and DTR (c). Black dots mark areas where either trends or correlations are significant at the $p < 0.05$ level.

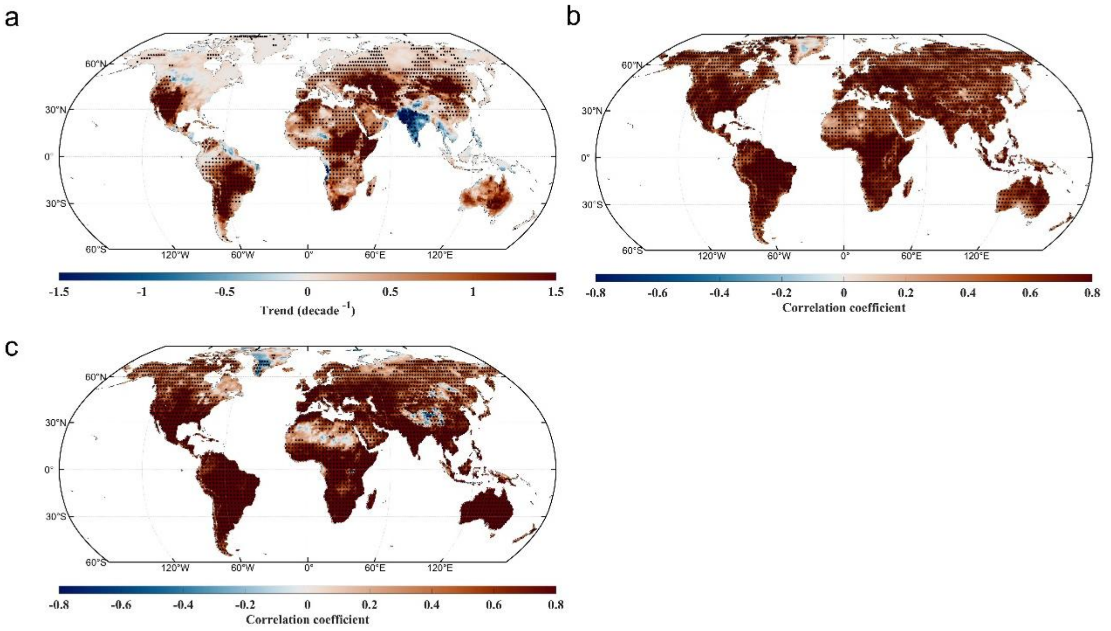

Supplementary Figure 23. Trend in the fire weather index (FWI) and its correlation with VPD and DTR during 1980–2023. a, Spatial distribution of the trend in FWI over global land areas. **b–c,** Spatial distribution of the Pearson correlation between FWI and VPD (b) and between FWI and DTR (c). Black dots mark areas where either trends or correlations are significant at the $p < 0.05$ level.

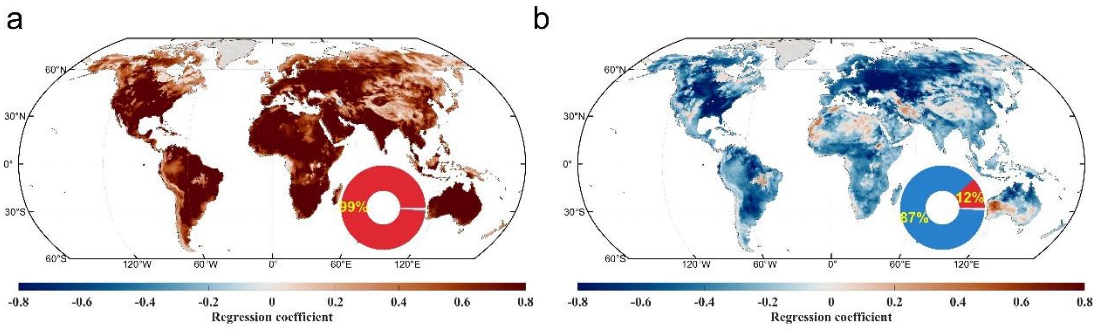

Supplementary Figure 24. Impact of daily maximum (T_{max}) and minimum temperatures (T_{min}) on vapor pressure deficit (VPD) on the interannual scale during 1980–2023, accounting for the influence of evapotranspiration (ET). Spatial distribution of RR coefficients of detrended annual AVP with respect to T_{max} (**a**) and T_{min} (**b**) based on ERA5-Land data, derived from a ridge regression (RR) model defined in: VPD ~ f (T_{max}, T_{min}, ET). Pie charts show the percentage of land area with positive (red), negative (blue), and non-significant (light grey) RR coefficients. "Non-significant" refers to cases where none of the coefficients in the RR model are statistically significant.

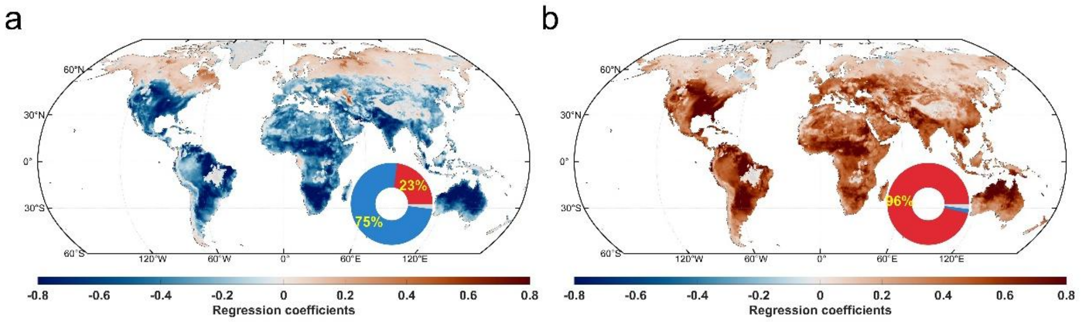

Supplementary Figure 25. Impact of daily maximum (T_{max}) and minimum temperatures (T_{min}) on actual vapor pressure (AVP) on the interannual scale during 1980–2023, accounting for the influence of evapotranspiration (ET). Spatial distribution of RR coefficients of detrended annual AVP with respect to T_{max} (**a**) and T_{min} (**b**) based on ERA5-Land data, derived from a ridge regression (RR) model defined in: AVP ~ f (T_{max}, T_{min}, ET). Pie charts show the percentage of land area with positive (red), negative (blue), and non-significant (light grey) RR coefficients. "Non-significant" refers to cases where none of the coefficients in the RR model are statistically significant.

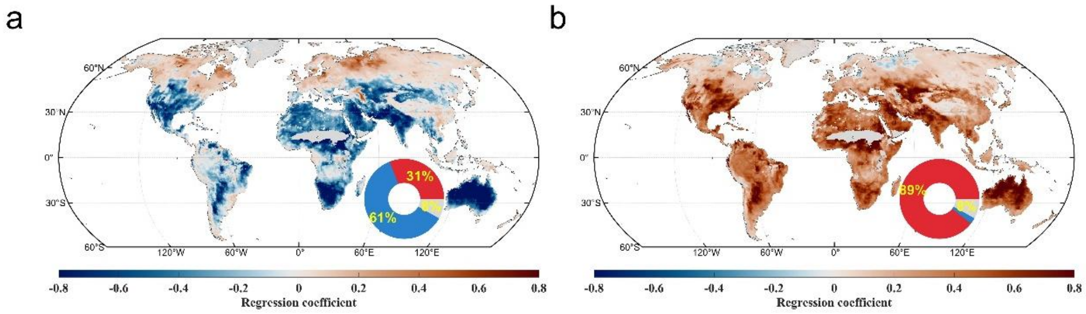

Supplementary Figure 26. Impact of daily maximum (T_{max}) and minimum temperatures (T_{min}) on actual vapor pressure (AVP) on the interannual scale during 1980–2023, accounting for the influence of surface incoming solar radiation (RS). Spatial distribution of RR coefficients of detrended annual AVP with respect to T_{max} (**a**) and T_{min} (**b**) based on ERA5-Land data, derived from a ridge regression (RR) model defined in: AVP ~ f(T_{max}, T_{min}, SM, RS), where SM is soil moisture. Pie charts show the percentage of land area with positive (red), negative (blue), and non-significant (light grey) RR coefficients. "Non-significant" refers to cases where none of the coefficients in the RR model are statistically significant.

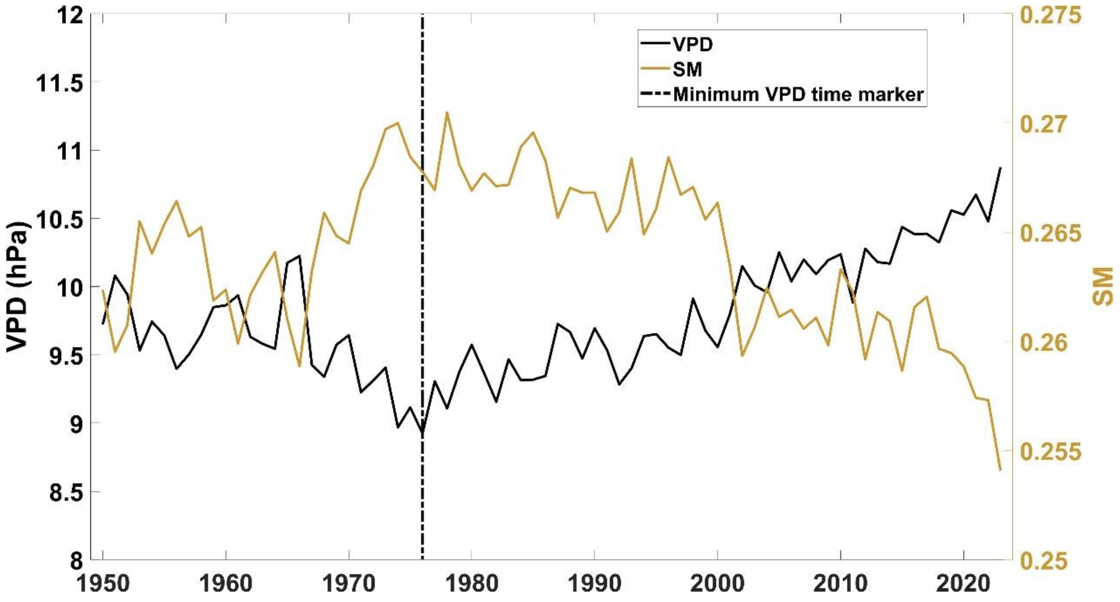

Supplementary Figure 27. Variations and trends in annual average vapor pressure deficit (VPD) and soil moisture (SM) over land areas from 1950 to 2023, based on the ERA5-Land dataset.

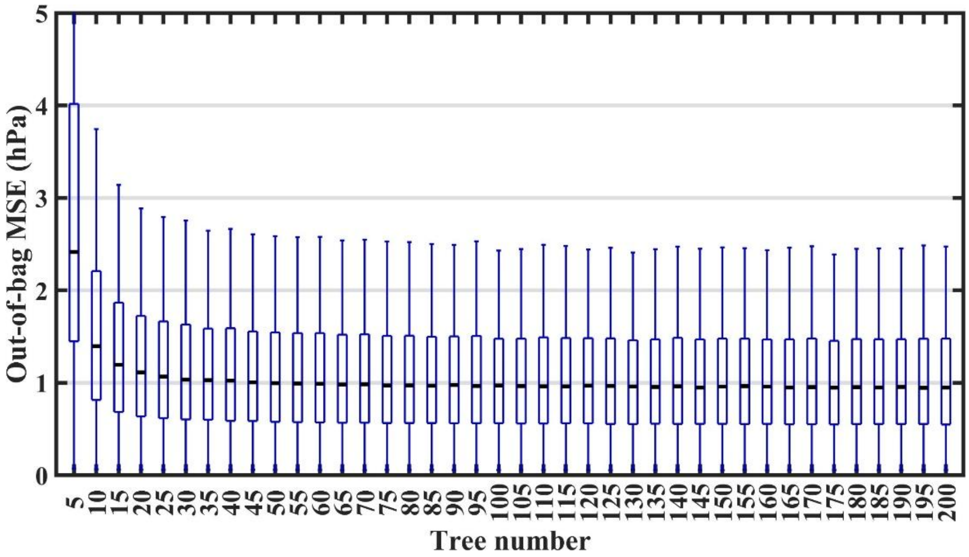

Supplementary Figure 28. Change in out-of-bag mean squared error (MSE) of the random forest regression (RF) regression model with varying numbers of decision trees across 1398 stations. The RF regression model is defined in Equation (2) and is based on monthly data. The height of each box represents the interquartile range, with the thick black line indicating the median and the box edges denoting the first and third quartiles. Whiskers extend to the 2.5th and 97.5th percentiles.

Supplementary References

1 Dunn, R. J. H., Willett, K. M., Parker, D. E. & Mitchell, L. Expanding HadISD: quality-controlled, sub-daily station data from 1931. *Geosci. Instrum. Method. Data Syst.* **5**, 473-491, doi:10.5194/gi-5-473-2016 (2016).

2 Muñoz-Sabater, J. *et al.* ERA5-Land: a state-of-the-art global reanalysis dataset for land applications. *Earth Syst. Sci. Data* **13**, 4349-4383, doi:10.5194/essd-13-4349-2021 (2021).

3 Dai, A. The diurnal cycle from observations and ERA5 in surface pressure, temperature, humidity, and winds. *Climate Dynamics* **61**, 2965-2990, doi:10.1007/s00382-023-06721-x (2023).

4 Dai, A. The diurnal cycle from observations and ERA5 in precipitation, clouds, boundary layer height, buoyancy, and surface fluxes. *Climate Dynamics* **62**, 5879-5908, doi:10.1007/s00382-024-07182-6 (2024).

5 Fatolahzadeh Gheysari, A., Maghoul, P., Ojo, E. R. & Shalaby, A. Reliability of ERA5 and ERA5-Land reanalysis data in the Canadian Prairies. *Theoretical and Applied Climatology* **155**, 3087-3098, doi:10.1007/s00704-023-04771-z (2024).

6 Almeida, M. & Coelho, P. A first assessment of ERA5 and ERA5-Land reanalysis air temperature in Portugal. *International Journal of Climatology* **43**, 6643-6663, doi:https://doi.org/10.1002/joc.8225 (2023).

7 Zou, J. *et al.* Performance of air temperature from ERA5-Land reanalysis in coastal urban agglomeration of Southeast China. *Science of The Total Environment* **828**, 154459, doi:https://doi.org/10.1016/j.scitotenv.2022.154459 (2022).

8 Zhao, P., He, Z., Ma, D. & Wang, W. Evaluation of ERA5-Land reanalysis datasets for extreme temperatures in the Qilian Mountains of China. *Frontiers in Ecology and Evolution* **11**, 1135895 (2023).

9 Zhao, P. & He, Z. A first evaluation of ERA5-Land reanalysis temperature product over the Chinese Qilian Mountains. *Frontiers in Earth Science* **10**, 907730 (2022).

10 Clelland, A. A., Marshall, G. J. & Baxter, R. Evaluating the performance of key ERA-Interim, ERA5 and ERA5-Land climate variables across Siberia. *International Journal of Climatology* **44**, 2318-2342, doi:https://doi.org/10.1002/joc.8456 (2024).

11 Liu, R. *et al.* Global-scale ERA5 product precipitation and temperature evaluation. *Ecological Indicators* **166**, 112481, doi:https://doi.org/10.1016/j.ecolind.2024.112481 (2024).

12 Bell, B. *et al.* The ERA5 global reanalysis: Preliminary extension to 1950. *Quarterly Journal of the Royal Meteorological Society* **147**, 4186-4227, doi:https://doi.org/10.1002/qj.4174 (2021).

13 Lin, J. *et al.* Outstanding performance of ERA5 reanalysis temperature in China since the 1950s and quantification of its abnormal error in 1965–1966. *Quarterly Journal of the Royal Meteorological Society* **n/a**, e5042, doi:https://doi.org/10.1002/qj.5042 (2025).
